# Perilipin2 plays a positive role in adipocytes during lipolysis by escaping proteasomal degradation

**DOI:** 10.1038/srep20975

**Published:** 2016-02-15

**Authors:** Yu Takahashi, Akihiro Shinoda, Haruhiko Kamada, Makoto Shimizu, Jun Inoue, Ryuichiro Sato

**Affiliations:** 1 Department of Applied Biological Chemistry, Graduate School of Agricultural and Life Sciences, The University of Tokyo, Tokyo, Japan; 2 Laboratory of Biopharmaceutical Research, National Institute of Biomedical Innovation, Osaka, Japan; 3 The Center for Advanced Medical Engineering and Informatics, Osaka University, Osaka, Japan

## Abstract

Perilipin2 (Plin2), also known as adipose differentiation-related protein (ADRP), or adipophilin, is a member of the PAT family involved in lipid droplet (LD) formation in the liver and peripheral tissues. Although Plin2 was originally identified as a highly expressed gene in adipocytes, its physiological role in mature adipocytes is largely unknown. In this report, we investigated the regulation of Plin2 expression and its function in differentiated adipocytes of mouse embryonic fibroblasts (MEFs). Plin2 mRNA levels increased during adipocyte differentiation whereas protein levels did not. Plin2 was degraded through the ubiquitin-proteasome pathway but was inhibited by lipolytic inducers. Furthermore, lentiviral-mediated Plin2 knockdown attenuated lipolysis in differentiated MEFs in a time-dependent manner. Oleic acid-induced LD formation enhanced Plin2 protein stability when it was localized to LDs. Furthermore, a mutational analysis revealed that the ubiquitination and degradation of Plin2 required both the second and third alanine in the N-terminal region. These results suggest that Plin2 is degraded in the cytosol in its N-terminal amino acid sequence-dependent manner and instead becomes stable when localized on LDs. Our findings highlight the relationship between protein stability and a previously unnoticed function of Plin2 during lipolysis in adipocytes.

Adipocyte hypertrophy in visceral fat is closely linked to the onset of metabolic syndrome^1^,^2^. Adipocytes function not only as the main reservoir of triacylglycerol (TAG) for an energy source but also as endocrine cells which secrete various cytokines called adipokines^3^. An increase of adipocyte size results in a change of the adipokine secretion: for example, an increase of the tumor necrosis factor α and a decrease of adiponectin, which exacerbates insulin resistance^4^.

Lipid droplets (LDs) comprise neutral lipids, including TAG, surrounded by a monolayer mainly consisting of phospholipids^5^. Many proteins have been identified that exist on the surface of LDs^6^,^7^,^8^. Among them, PAT family members including Plin1/perilipin, Plin2/ADRP, Plin3/tail-interacting 47 kDa protein, Plin4/S3–12, and Plin5/myocardial LD protein (MLDP) play a pivotal role in the formation and/or degradation of LDs^9^,^10^. An adipocyte maturation model suggests that PAT family proteins localize on and stabilize LDs depending on their size^11^. For instance, Plin1, which is predominantly expressed in adipocytes, is one of the target genes of peroxisome proliferator-activated receptor γ (PPARγ)^12^, a master regulator of adipogenesis, can localize on larger LDs during adipocyte differentiation through the replacement of Plin2. Plin1 deficient mice show a lean phenotype and are resistant to diet-induced obesity, accompanied by higher basal lipolytic activities^13^,^14^.

Lipolysis is a physiological phenomenon in which TAG is hydrolyzed into glycerol and free fatty acids (FFAs) in response to energy demands such as fasting^15^. FFAs circulate in the blood and are provided to peripheral tissues as an energy source. Various factors have been found to be involved in the lipolytic pathway. Briefly, once β-adrenergic receptors are activated by their ligands such as catecholamine’s, cyclic adenosine monophosphate (cAMP) levels are induced and protein kinase A is subsequently activated; Plin1 and hormone sensitive lipase are then both phosphorylated, which ultimately leads to the hydrolysis of TAG^9^,^15^. Adipose triglyceride lipase (ATGL) and its activator, comparative gene identification-58 (CGI-58), are also critical regulators of the initial step of lipolysis^16^,^17^. However, the molecular mechanisms involved in the lipolytic cascade remain unknown. For example, the regulation of intracellular CGI-58 localization in response to lipolytic stimuli.

Plin2 is ubiquitously expressed and also reported to be involved in LD formation^18^,^19^. It is induced in the liver during fasting^20^ and contributes to the development of fatty liver diseases, which has been proven by knockout mice experiments^21^, although the mice still produce an N-terminally truncated form of Plin2, which possesses biological activities^22^. Recent studies have shown that Plin2 deficient mice without any truncated products are protected against adipose inflammation and the development of insulin resistance, followed by a decrease of FFA concentrations in the blood^23^. However, although Plin2 was initially identified as a highly expressed gene in adipocytes, its main role in adipocytes remains largely unclarified.

The ubiquitin-proteasome system plays a critical role in protein degradation in eukaryotic cells for its quality control^24^,^25^. A large number of proteins are subjected to the system and its dysregulation is linked to the pathogenesis of diseases such as cancer and neurodegeneration. Plin2 has also been reported as a substrate of 26S proteasome^26^,^27^ and the detailed regulation in adipocytes has not been fully investigated.

In this study, to elucidate the precise roles of Plin2 in adipocytes, we investigated the mechanism regarding its expression and function during LD formation using either an adipocyte differentiation model or a simplified LDs formation model by loading bovine serum albumin (BSA)-conjugated oleic acids (OAs) on non-adipogenic cells. We found that the Plin2 was stabilized upon lipolytic stimuli and subsequently contributed to the enhancement of lipolysis. Furthermore, while the Plin2 in the LDs fraction was protected from protein degradation, cytosolic Plin2 was susceptible to ubiquitin-proteasomal degradation.

## Results

### Plin2 mRNA levels but not protein levels are increased during adipogenesis

**Figure 1:**
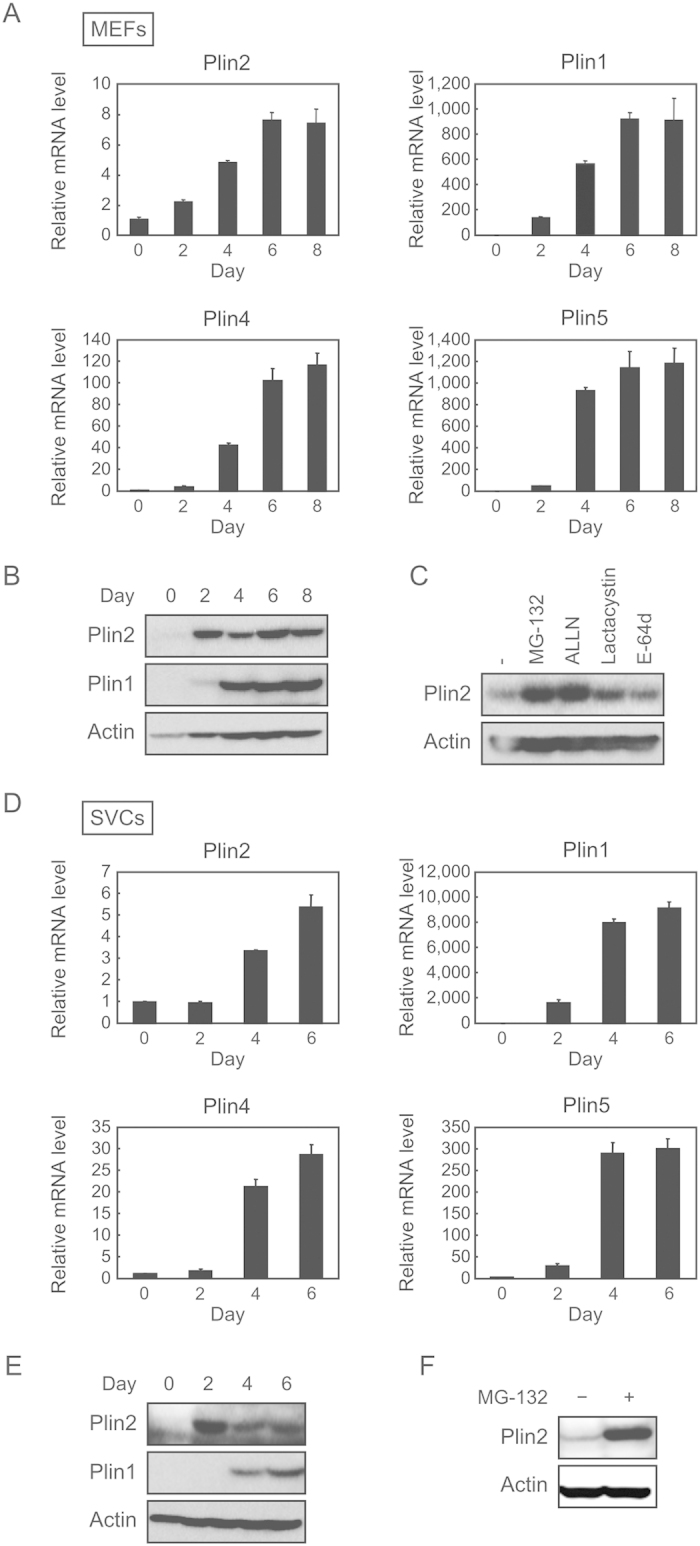
Plin2 mRNA but not protein levels are increased during adipocyte differentiation of MEFs and SVCs. (**A**) MEFs were subjected to adipocyte differentiation as described in the Materials and Methods and the cells were harvested every 2 days. Quantitative RT-PCR was performed and each mRNA level was determined and normalized by 18s rRNA levels. These data represent the means ± S.D. The assay was performed in triplicate. (**B**) Differentiated MEFs were harvested every 2 days and each protein level was determined using Western blot analysis. (**C**) After 7 days of differentiation, MEFs were treated with 10 μM MG-132, 50 μM ALLN, 10 μM Lactacystin, or 10 μM E-64d for 12 h. Plin2 and actin proteins from whole cell extracts were detected using Western blot analysis. (**D**) SVCs were subjected to adipocyte differentiation as described in the Materials and Methods and cells were harvested every 2 days. Quantitative RT-PCR was performed and each mRNA level was determined and normalized by 18s rRNA levels. These data represent the means ± S.D. The assay was performed in triplicate. (**E**) Differentiated SVCs were harvested every 2 days and each protein level was determined using Western blot analysis. (**F**) After 7 days of differentiation, SVCs were treated with 10 μM MG-132 for 12 h. Plin2 and actin proteins from whole cell extracts were detected using Western blot analysis.

We and other groups have previously shown that 3T3-L1 cells, which are a widely used model for adipogenesis studies, do not express some important genes which are expressed *in vivo* white adipose tissues (WATs), such as estrogen receptor α (ERα)^28^, Plin5^20^, and sterol regulatory element binding protein-1c (SREBP-1c)^29^. We have also shown that these genes are expressed in adipocyte-differentiated mouse embryonic fibroblasts (MEFs) or stromal vascular cells (SVCs)^30^. Taking these into consideration, we regard MEFs and SVCs as more physiological adipogenesis models and decided to use them in order to elucidate the physiological role of Plin2. At first, we investigated mRNA and protein levels of Plin2 in MEFs. The Plin2 mRNA expression was induced during adipocyte differentiation of MEFs as well as Plin1, Plin4, and Plin5 which are other members of the PAT family (Fig. 1A). On the other hand, Plin2 protein levels were not induced during adipogenesis, whereas Plin1 protein drastically increased (Fig. 1B). We obtained similar data using SVCs; that is while both Plin2 and Plin1 mRNA levels increased during adipogenesis (Fig. 1D), Plin2 protein levels did not (Fig. 1E). These results are consistent with previous reports using 3T3-L1 cells^18^.

Since Plin2 can be degraded through the ubiquitin-proteasome pathway^26^,^27^, we investigated whether Plin2 expressed in MEF adipocytes is subjected to proteasomal degradation using various inhibitors. We found that MG-132 and N-Acetyl-Leu-Leu-Norleu-al (ALLN), both of which are 26S proteasome inhibitors, substantially increased Plin2 protein levels in differentiated MEFs. However, lactacystin, a 20S proteasome inhibitor, and E-64d, a cysteine protease inhibitor, only slightly increased its protein levels (Fig. 1C). It was shown that Plin2 in differentiated SVCs also increased with the treatment of MG-132 (Fig. 1F). These results suggest that the Plin2 is degraded through the 26S proteasome pathway during adipogenesis in both MEFs and SVCs, and the reason why Plin2 protein levels did not increase during adipogenesis may be attributed to the susceptibility of protein degradation.

Although we consider that SVCs are a faithful adipogenesis model as well as MEFs, the number of cells obtained from a single mouse is rather small and performable experiments are limited. Therefore, we mainly used MEFs in the following experiments.

### Plin2 is increased by lipolytic inducers and stimulates lipolysis in differentiated MEFs

**Figure 2:**
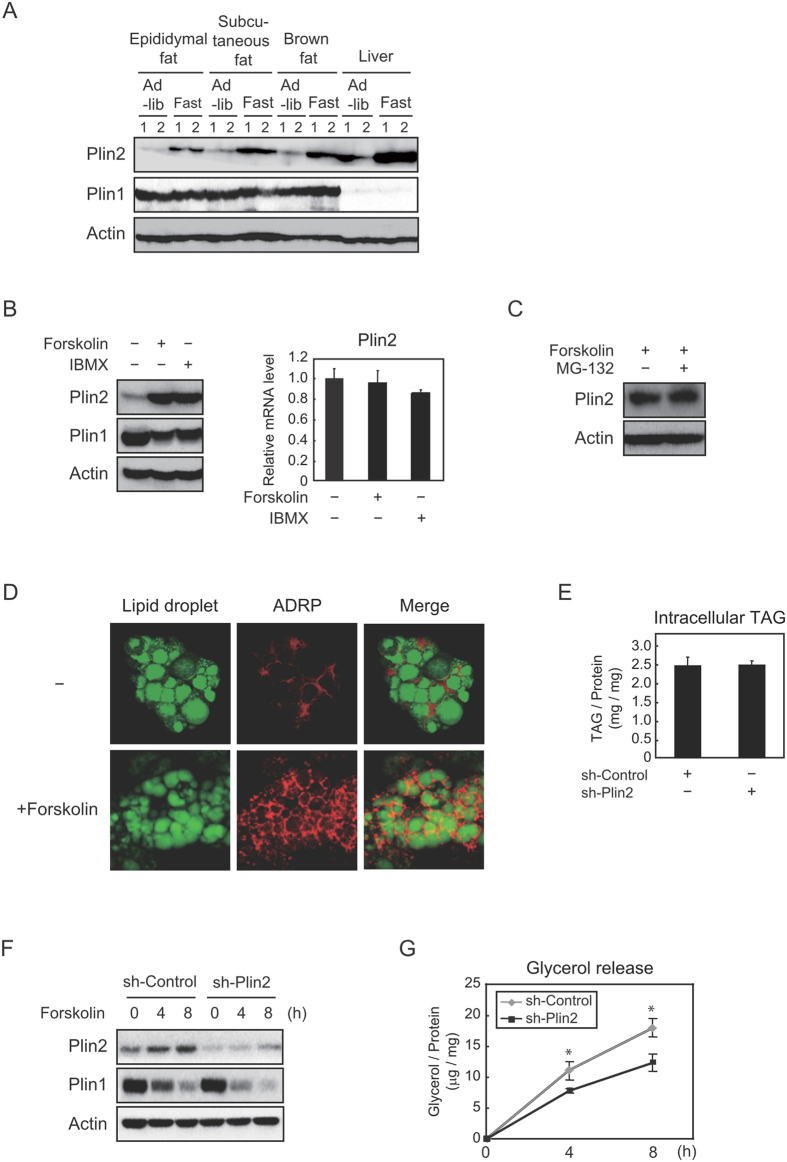
Plin2 is stabilized at the protein level by lipolytic stimuli and enhances lipolysis in differentiated MEFs. (**A**) Total proteins were extracted from epididymal fat, subcutaneous fat, brown fat, and liver of 9-weeks-old C57BL/6J mice fed ad libitum or fasted for 24 h (n = 4, 2 samples were pooled each). Each protein level was determined using Western blot analysis. (**B**) MEFs differentiated into adipocytes for 6 days were treated with 10 μM forskolin or 500 μM IBMX for 12 h. Each protein level from whole cell extracts was determined using Western blot analysis (left). The Plin2 mRNA level was determined using quantitative RT-PCR and normalized to 18s rRNA levels. These data represent the means ± S.D. The assay was performed in triplicate (right). (**C**) After 7 days of adipocyte differentiation, MEFs were treated with 10 μM forskolin together with or without 10 μM MG-132 for 12 h. Plin2 and actin proteins from whole cell extracts were detected using Western blot analysis. (**E**) MEFs differentiated into adipocytes for 8 days were treated with 10 µM forskolin for 24 h. The cells were stained with BODIPY 493/503 (green, LDs) and anti-Plin2 antibody (red), and then underwent confocal image analysis as described in the Material and Methods. (**D**) Two days before they were subjected to adipocyte differentiation, MEFs were infected with a lentiviral vector expressing shRNA for either the control or Plin2. Intracellular TAG was quantified using the Triglyceride E-test Wako from MEFs after 8 days of adipocyte differentiation. These data represent the means ± S.D. The assay was performed in triplicate. (**F**,**G**) MEFs were infected with either an sh-control or an sh-Plin2 expressing lentiviral vector as in (**E**). After 8 days of adipocyte differentiation, the cells were treated with 10 μM forskolin for 4 h or 8 h. (**F**) Each protein level from whole cell extracts were determined using Western blot analysis. (**G**) The supernatants and cells were separately collected. The amounts of released glycerol were normalized to cellular protein levels, both of which were determined as given in Materials and Methods. These data represent the means ± S.D. The assay was performed in triplicate. *P < 0.01.

It has been reported that Plin2 protein levels are induced during fasting in the liver^20^. Hence, we verified if the phenomenon could be also observed in adipose tissues. It was found that when mice were subjected to fasting, Plin2 expression in epididymal fat, subcutaneous fat and brown fat was induced as well as in the liver (Fig. 2A). On the other hand, Plin1 expression was not largely affected in any fat tissues. Considering that fasting induces physiological lipolysis in WATs in order to supply FFAs to peripheral tissues, we speculated that lipolytic stimuli affected Plin2 expression. Thus, we investigated the change of Plin2 expression under lipolytic conditions *in vitro* induced by an adenylyl cyclase activator, forskolin, and a phosphodiesterase inhibitor, 3-Isobutyl-1-methylxanthine (IBMX), both of which increase intracellular cAMP levels. When differentiated MEFs were treated with these inducers, Plin2 protein levels dramatically increased (Fig. 2B, left), whereas Plin2 mRNA levels remained unaffected (Fig. 2B, right). In addition, when forskolin-treated differentiated MEFs were further treated with a proteasome inhibitor MG-132, Plin2 protein levels did not increase (Fig. 2C), which indicates that lipolytic stimuli attenuated Plin2 protein degradation through a ubiquitin-proteasome pathway. Moreover, we found that while Plin2 mainly localized in the cytoplasm space before lipolytic stimulation, it was strongly stained around the LDs after the stimulation (Fig. 2D).

Furthermore, we performed an Plin2 knockdown experiment by lentiviral-mediated shPlin2, and found that intracellular TAG accumulation remained largely unchanged (Fig. 2E). We next investigated whether Plin2 knockdown affects lipolysis using these cells. Before lipolytic stimulation using forskolin, we confirmed a substantial decrease in Plin2 protein and almost the same levels of Plin1 protein by the knockdown of Plin2 (Fig. 2F). Surprisingly, we observed that the glycerol release was statistically decreased by Plin2 knockdown after 4 h and 8 h of the lipolytic stimuli (Fig. 2G), demonstrating that Plin2 increased lipolysis and did not protect LDs from the lipolytic enzyme attacks. Notably, the degrees of decrease in the Plin1 protein during lipolysis were somewhat greater in the sh-Plin2 group (Fig. 2F). These results suggest that Plin2 is induced at protein levels by lipolytic stimuli and translocates from cytosol to LDs, thereby functioning to increase lipolytic activities, not TAG accumulation in differentiated MEFs.

### Plin2 protein levels are increased in differentiated Plin1-deficient MEFs

**Figure 3:**
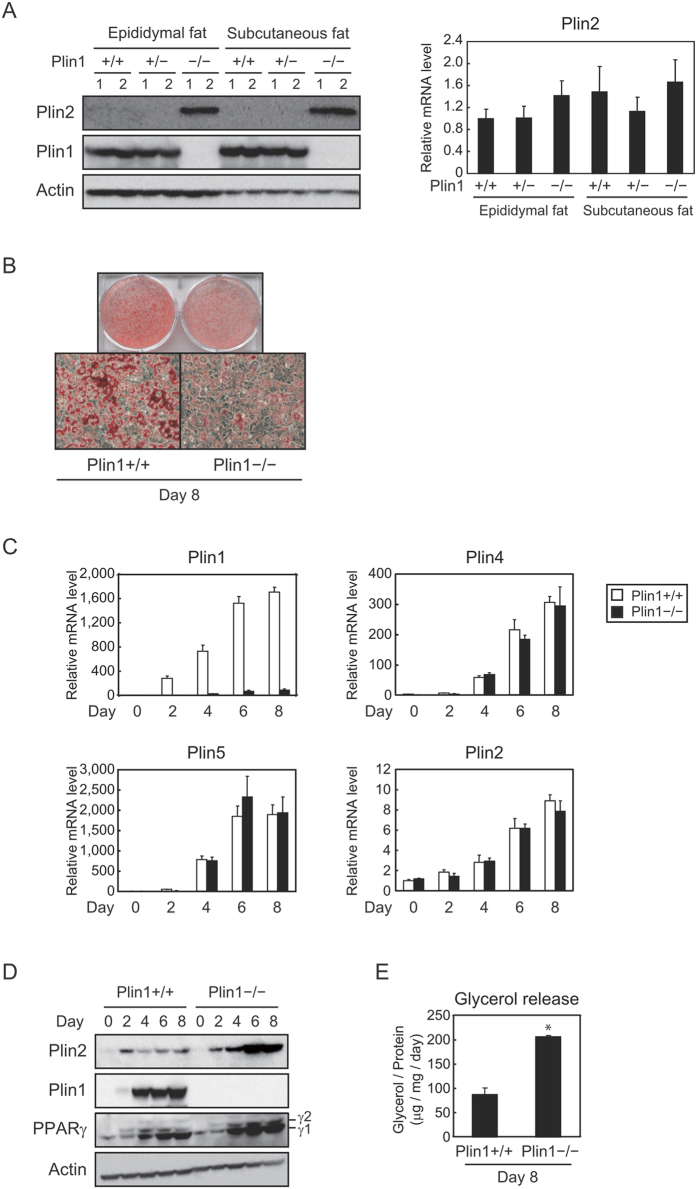
Protein levels of Plin2 are increased during adipocyte differentiation of Plin1 deficient MEFs. (**A**) Epididymal and subcutaneous fat pads were isolated from 10–14 weeks-old Plin1+/+, Plin1+/− or Plin1−/− mice fed ad libitum (n = 6, 3 samples were pooled each). Each protein level was detected using Western blot analysis (left) and the Plin2 mRNA level was determined using quantitative RT-PCR and normalized to 18s rRNA levels (right). These data represent the means ± S.D. The assay was performed in triplicate. (**B**) Differentiated (day 8) MEFs obtained from Plin1+/+ or Plin1−/− mice were stained with Oil Red O. (**C**) Plin1+/+ and Plin1−/− MEFs were differentiated into adipocytes and harvested every 2 days. Each mRNA level was determined using quantitative RT-PCR and normalized to 18s rRNA levels. These data represent the means ± S.D. The assay was performed in triplicate. (**D**) Plin1+/+ or Plin1−/− MEFs were differentiated into adipocytes and harvested every 2 days. Each protein level was detected using Western blot analysis. (**E**) After 8 days of differentiation, glycerol levels contained in the supernatant were determined and normalized to total cellular protein levels as in the Materials and Methods. These data represent the means ± S.D. The assay was performed in triplicate. *P < 0.01.

It has been reported that Plin1 knockout mice show a lean phenotype and Plin2 protein levels are increased in WATs compared with wild-type mice^13^. We independently confirmed that Plin2 protein levels were increased in the epididymal and subcutaneous fat of Plin−/− mice compared with Plin1+/+ and Plin1+/− mice (Fig. 3A, left); although Plin2 mRNA levels were comparable among all genotypes (Fig. 3A, right). In order to furtherly investigate the molecular mechanism of Plin2 expression *in vitro*, we prepared MEFs from Plin1+/+ or Plin1−/− mice and then differentiated them into adipocytes. As we have shown previously^31^, LD formation was obviously suppressed in Plin1−/− MEF adipocytes (Fig. 3B). However, mRNA levels of Plin4, Plin5, and Plin2 were induced at similar levels during adipogenesis of both genotypes (Fig. 3C). Considering that Plin4 and Plin5 are target genes of PPARγ in adipocytes^30^,^32^, differentiating capacities were confirmed to be almost the same between both genotypes. In addition, protein levels of PPARγ during adipogenesis of Plin1+/+ and Plin1−/− MEFs were comparable to each other (Fig. 3D). Interestingly, Plin2 expression at the protein level was drastically induced in Plin1−/− MEFs but not in Plin1+/+ MEFs (Fig. 3D). Besides this, the glycerol release which reflects lipolysis was augmented in adipocytes from Plin1−/− MEFs rather than Plin1+/+ MEFs (Fig. 3E), which was consistent with the *in vivo* tissue analysis^13^, and also supports the notion that Plin2 enhanced lipolytic activities (Fig. 2G).

### Plin2 escapes from proteasome degradation in differentiated Plin1-deficient MEFs

**Figure 4:**
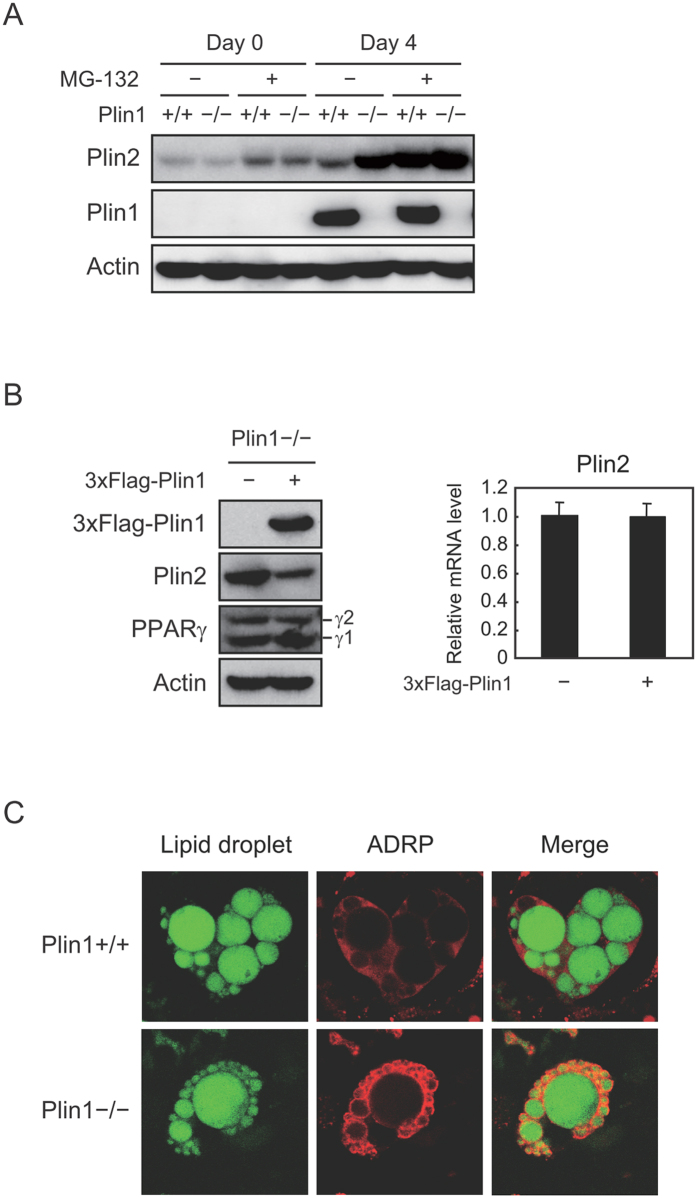
Plin2 in adipocytes from Plin1−/− MEFs escapes from proteasome degradation and localizes on LDs. (**A**) Undifferentiated (day 0) and differentiated (day 4) MEFs from Plin1+/+ or Plin1−/− were treated with 10 μM MG-132 for 12 h. Each protein level from whole cell extracts was detected using Western blot analysis. (**B**) Plin1−/− MEFs infected with either a mock or a 3×Flag-Plin1 retroviral expression vector were differentiated into adipocytes for 6 days. Each protein level from whole cell extracts was detected using Western blot analysis (left). The Plin2 mRNA level was determined using quantitative RT-PCR and normalized to 18s rRNA levels. These data represent the means ± S.D. The assay was performed in triplicate (right). (**C**) Plin1+/+ or Plin1−/− MEFs were differentiated into adipocytes for 8 days. The cells were stained with BODIPY 493/503 (green, LDs) and anti-Plin2 antibody (red), and then underwent confocal image analysis as described in the Material and Methods.

We further investigated the mechanism for an increase in Plin2 protein in differentiated Plin1−/− MEFs. As expected from Fig. 1C, Plin2 protein levels of both undifferentiated and adipocyte-differentiated Plin1+/+ MEFs were increased by the treatment of MG-132 (Fig. 4A). On the other hand, the protein levels of Plin1−/− MEFs were as high as that of MG-132-treated Plin1+/+ MEFs, and did not further increase by the addition of MG-132 (Fig. 4A). On top of that, retroviral-mediated 3×Flag-tagged Plin1 overexpression reversed the phenotype of Plin2 increase in Plin1−/− MEF adipocytes (Fig. 4B, left). It should be noted that neither PPARγ protein nor Plin2 mRNA levels were affected by the exogenous Plin1 expression (Fig. 4B, left and right). The results clearly show that the increase in Plin2 protein in differentiated Plin1−/− MEFs was due to a Plin1 deficiency and subsequent protein stabilization. Furthermore, although Plin2 was ubiquitously stained in the cytoplasm space in Plin1+/+ MEFs, it localized around the LDs in Plin1−/− MEFs (Fig. 4C). From these data, we conclude that the phenotype of increased Plin2 protein levels in Plin1−/− adipose tissues was due to its protein stabilization, rather than a compensatory increased expression caused by a Plin1 deficiency. Taken together, it is plausible that LDs surrounded by Plin2 become susceptible to lipolysis, which may contribute to the appearance of another phenotype of Plin1 knockout mice exhibiting elevated basal lipolytic activities.

### Plin2 is stabilized when localized to LDs

**Figure 5:**
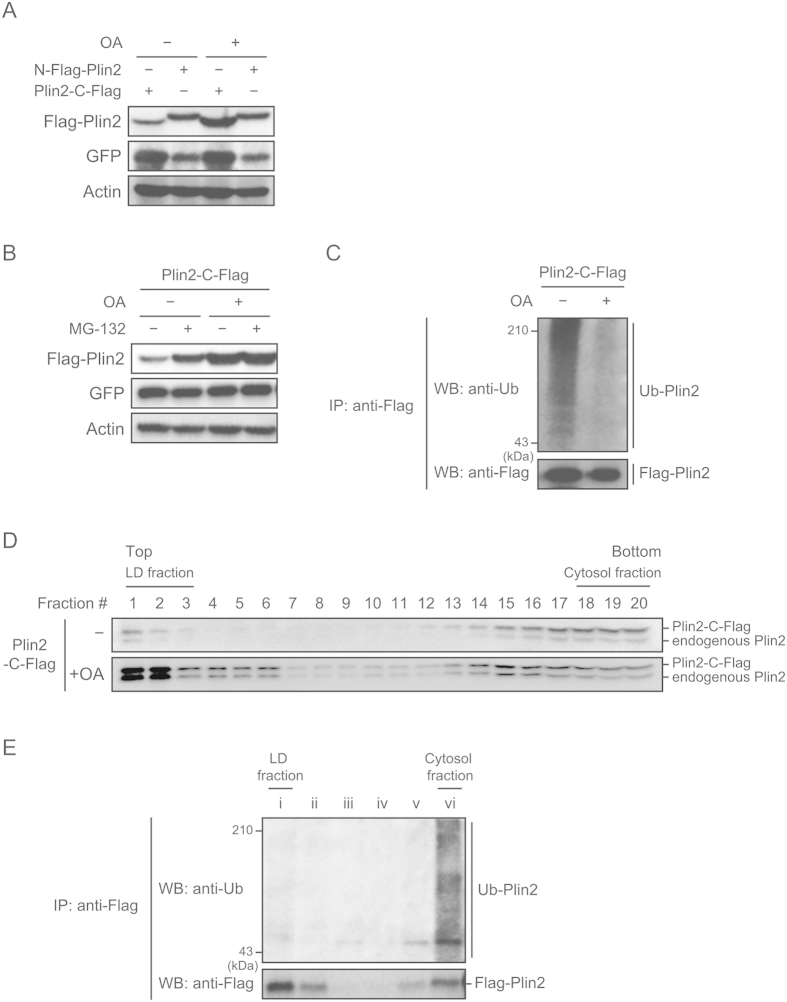
Localization on LDs stabilizes Plin2 in BSA-conjugated OA-treated cells. (**A**) NIH-3T3 cells infected with either an N-Flag-Plin2 or an Plin2-C-Flag lentiviral expression vector were treated with 500 μM BSA-conjugated OAs for 24 h. Each protein level from whole cell extracts was detected using Western blot analysis with anti-Flag, anti-GFP, and anti-actin antibodies. (**B**) NIH-3T3 cells infected with a C-Flag-Plin2 lentiviral expression vector were treated with 500 μM BSA-conjugated OAs for 24 h, followed by a treatment of 10 μM MG-132 for 12 h. Each protein level from whole cell extracts was detected using Western blot analysis with anti-Flag, anti-GFP, and anti-actin antibodies. (**C**) NIH-3T3 cells infected with a C-Flag-Plin2 lentiviral expression vector were treated with 500 μM BSA-conjugated OAs for 24 h, followed by a treatment of 10 μM MG-132 for 12 h. Whole cell lysates were subjected to immunoprecipitation with the anti-Flag antibody. The precipitates were then processed using Western Blot analysis with anti-ubiquitin and anti-Flag antibodies. (**D**) NIH-3T3 cells infected with a C-Flag-Plin2 lentiviral expression vector were treated with 500 μM BSA-conjugated OAs for 24 h. The supernatants of cell homogenates were then subjected to a sucrose density gradient to obtain LD fractions as described in the Material and Methods. An equal aliquot of each fraction was subjected to Western blot analysis with the anti-Plin2 antibody. Upper bands indicate C-Flag-Plin2 and lower bands indicate endogenous Plin2. (**E**) NIH-3T3 cells infected with a C-Flag-Plin2 lentiviral expression vector were treated with 500 μM BSA-conjugated OAs for 24 h and then subjected to subcellular fractionation as in (**D**). An equal aliquot of each fraction was processed to immunoprecipitation with anti-Flag antibody and a subsequent Western blot analysis with anti-ubiquitin and anti-Flag antibodies.

**Figure 6:**
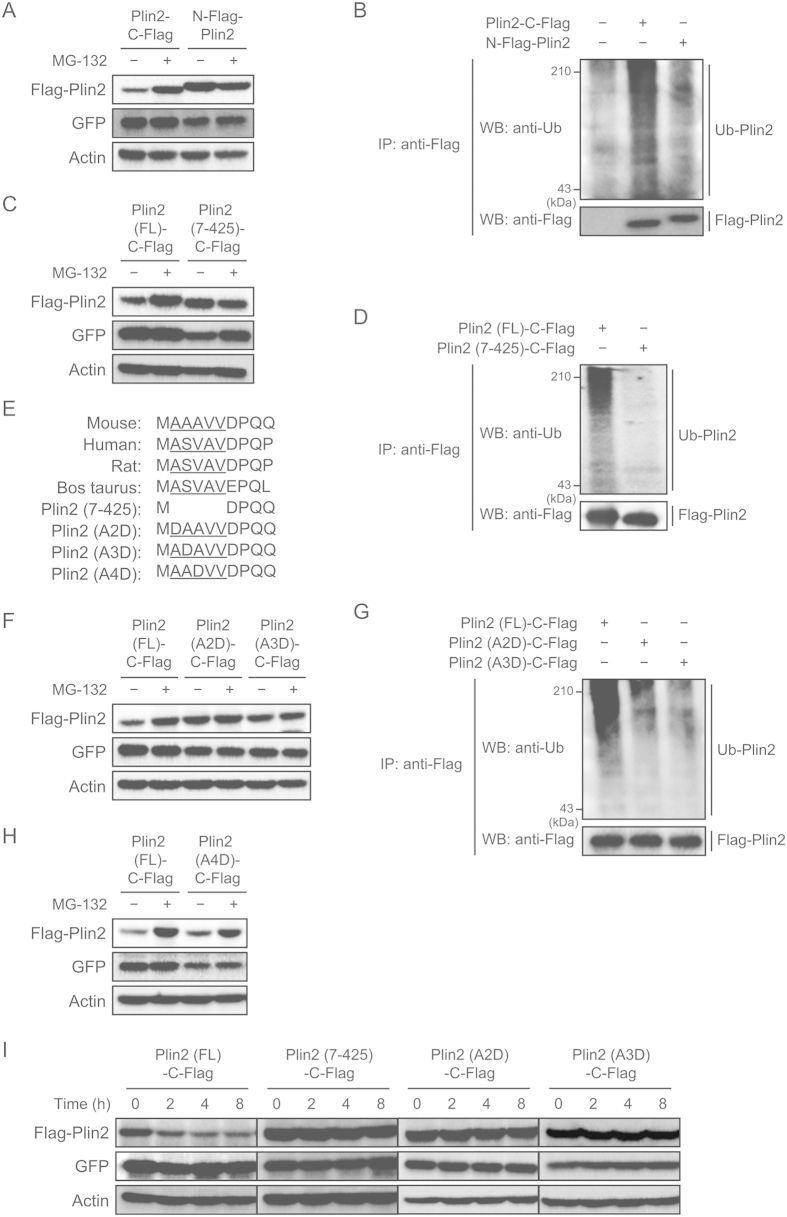
Second and third alanine residues are essential for protein degradation of Plin2. (**A**,**B**) NIH-3T3 cells infected with either an N-Flag-Plin2 or an Plin2-C-Flag lentiviral expression vector were treated with 10 μM MG-132 for 12 h. (**A**) Each protein level from whole cell extracts was detected using Western blot analysis with anti-Flag, anti-GFP and anti-actin antibodies. (**B**) Whole cell lysates were subjected to immunoprecipitation with anti-Flag antibody. The precipitates were then processed to Western Blot analysis with anti-ubiquitin and anti-Flag antibodies. (**C**,**D**) NIH-3T3 cells infected with either an Plin2-C-Flag or a deletion mutant Plin2 (7–425)-C-Flag lentiviral expression vector were treated with 10 μM MG-132 for 12 h. (**C**) Whole cell lysates were subjected to Western blot analysis as in (**A**). (**D**) Whole cell lysates were subjected to immunoprecipitation and the following Western blot analysis as in (**B**). (**E**) N-terminal amino acid sequences of Plin2 are compared among species and Plin2 mutants. Underlines denote amino acids with small side chains. (**F**,**G**) NIH-3T3 cells infected with a lentiviral expression vector with Plin2-C-Flag, point mutant Plin2 (A2D)-C-Flag or point mutant Plin2 (A3D)-C-Flag were treated with 10 μM MG-132 for 12 h. (**F**) Whole cell lysates were subjected to Western blot analysis as in (**A**). (**G**) Whole cell lysates were subjected to immunoprecipitation and the following Western blot analysis as in (**B**). (**H**) NIH-3T3 cells infected with either an Plin2-C-Flag or a point mutant Plin2 (A4D)-C-Flag lentiviral expression vector were treated with 10 μM MG-132 for 12 h. Each protein level from whole cell extracts was detected using Western blot analysis with anti-Flag, anti-GFP, and anti-actin antibodies. (**I**) NIH-3T3 cells were infected with a lentiviral expression vector with Plin2-C-Flag, deletion mutant Plin2 (7–425)-C-Flag, point mutant Plin2 (A2D)-C-Flag, or point mutant Plin2 (A3D)-C-Flag and treated with 10 μg/mL cycloheximide for 30 min. The cells were harvested every two hours, and each protein level from whole cell extracts was detected using Western blot analysis with anti-Flag, anti-GFP and anti-actin antibodies.

We next investigated how Plin2 protein degradation is regulated during LD formation. To elucidate this, we adopted a simplified model using NIH-3T3 cells, which form LDs by adding BSA-conjugated OA into a culture medium in the absence of Plin1^33^. At first, we verified if exogenously expressed Plin2 increased after the addition of OA. We chose a lentiviral system to exogenously express both Plin2 and Venus, a modified green fluorescent protein (GFP). We used the Venus protein as an internal control to monitor infection efficiency since the expression system uses an internal ribosome entry site (IRES), therefore, mRNA expression of Venus reflects that of Plin2. Interestingly, while the C-terminal Flag-tagged Plin2 (Plin2-C-Flag) expression was increased by the addition of OA, the N-terminal Flag-tagged Plin2 (N-Flag-Plin2) was not (Fig. 5A). The phenomenon that N-Flag-Plin2 was not degraded will be further investigated in Fig. 6. We next examined whether the increased Plin2 by OA was due to an escape from proteasome degradation. As expected, MG-132 stabilized exogenously expressed Plin2-C-Flag in an OA-untreated group, but not in an OA-treated group (Fig. 5B). An immunoprecipitation analysis also showed that ubiquitination levels of Plin2-C-Flag were obviously reduced by the OA treatment (Fig. 5C). Furthermore, we performed a subcellular fractionation analysis and revealed that Plin2 was mainly localized in the LD fraction after the OA treatment (Fig. 5D). Nonetheless, ubiquitination levels in the LD fraction were much lower than in the cytosol fraction (Fig. 5E). The results clearly indicate that Plin2 is subjected to ubiquitin-proteasome degradation in the cytosol, but not when it localizes to LDs.

### N-terminal alanine residues are critical for Plin2 protein degradation

We next investigated the mechanism as to which Plin2 N-terminal region was responsible for its degradation. First, we examined whether N-Flag-Plin2, the protein levels of which were not increased by the OA treatment, were susceptible to protein degradation or not. We found that while Plin2-C-Flag was stabilized by the MG-132 treatment as in Fig. 5B, N-Flag-Plin2 was not stabilized by the treatment (Fig. 6A). An immunoprecipitation analysis also clarified that ubiquitination was reduced in N-Flag-Plin2 compared with Plin2-C-Flag (Fig. 6B). Based on these results, we determined that the N-terminal region had an impact on Plin2 protein degradation. We then newly constructed a deletion mutant Plin2 (7–425)-C-Flag, which lacks the amino acid sequences from the second alanine to the sixth valine, and examined its susceptibility to protein degradation. The result showed that the mutant protein was not stabilized by the MG-132 treatment, suggesting that it was poorly degraded (Fig. 6C). Furthermore, the ubiquitination levels were much less than the full-length (FL) Plin2-C-Flag (Fig. 6D). Taken together, the N-terminal region is clearly critical for Plin2 ubiquitination and its subsequent degradation.

It has been reported that N-terminal residues are important for protein degradation through pathways called the N-end rule^34^,^35^,^36^. We then searched N-terminal amino acid sequences in various species and found that 2–6 residues in the N-terminal region were well conserved among species that possess amino acids with small side chains, such as alanine, valine, and serine (Fig. 6E). Therefore, we constructed Plin2 mutants with a single point mutation each and compared their protein degradation. Intriguingly, we observed that neither Plin2 (A2D)-C-Flag nor Plin2 (A3D)-C-Flag was stabilized by the MG-132 treatment (Fig. 6F). Also, these Plin2 mutants were less ubiquitinated than the Plin2 (FL)-C-Flag (Fig. 6G). It is interesting that the Plin2 (A4D)-C-Flag was stabilized after the MG-132 treatment as well as the Plin2 (FL)-C-Flag (Fig. 6H). These results clearly show that second and third alanine residues are both required for Plin2 protein degradation. Consistent with these data, a protein decay assay using cycloheximide that inhibits *de novo* protein synthesis revealed that while Plin2 (FL)-C-Flag was rapidly degraded, Plin2 mutants (7-425, A2D, A3D) were all stable after 8 h of the cycloheximide treatment (Fig. 6I).

## Discussion

In this study, we focused on the function and expression control of Plin2 during adipogenesis of MEFs, which we believe have good physiological characteristics closer to *in vivo* adipocytes. Although Plin2 was identified as a high expression gene in adipocytes, its role was considered to form smaller LDs before Plin1 expression was induced. We wondered whether the main role of Plin2 was restricted to the initial stage of adipocyte differentiation as suggested by an LD maturation model^11^, since Plin2 mRNA was induced long after the larger LDs were generated where Plin1 localized (Fig. 1A,D). Furthermore, we considered it essential to determine the experimental conditions in which Plin2 increased or stabilized in order to predict the physiological role of Plin2. As a result, we found that Plin2 increased due to escape from proteasome degradation once stimulated by lipolytic inducers (Fig. 2A,B) and thereby increased lipolysis as shown by a knockdown experiment (Fig. 2G). The results were also supported using Plin1 deficient MEFs stating that Plin2 protein levels were higher in differentiated adipocytes in Plin1−/− MEFs due to proteasome degradation (Figs 3D and 4A), whose lipolytic activities are higher than Plin1+/+ MEFs (Fig. 3E). This evidence accounts for the fact that Plin1 deficient mice exhibit higher lipolytic activities at a basal state^13^, which contributes to their lean phenotype. Also, it is noteworthy that these findings are consistent with a previous report, in which serum levels of FFAs in Plin2 deficient mice were significantly lower than that of wild-type mice^23^.

We also carried out a proteome analysis to identify Plin2 interacting proteins. We have so far identified several proteins that interact with exogenously expressed Plin2, such as the heat shock cognate protein of 70 kDa (Hsc70) (unpublished data). Hsc70 has already been reported to be localized on LDs and is also associated with Plin2^37^. We consider that some Plin2-associated proteins would coordinately function to regulate lipolytic activities and should be examined one-by-one in future studies. Moreover, besides promoting lipolysis through interaction with other proteins, Plin2 may disrupt the recruitment of other LD-associated proteins with LD-protective capacities. We suppose that such proteins could also be identified through proteome analysis by changing the experimental conditions. In addition, considering that Plin2 is reported to coat smaller LDs than Plin1, it may be possible that the surface area of LDs, and not that of Plin2 itself, regulates the recruitment of LD-associated proteins that promote lipolysis. Therefore, the comparison of whole LD surface proteins in MEFs may reveal the mechanism as to how lipolysis is induced once Plin1 is replaced by Plin2 on LDs.

Recently, it was reported that the knockdown of Plin2 did not affect lipolysis in 3T3-L1 cells^38^ while our research for this paper was under investigation. The 3T3-L1 cells, however, have several distinct characters from MEFs. For example, ERα and SREBP-1c are not expressed in 3T3-L1 adipocytes but are expressed in MEFs adipocytes and *in vivo* WATs^30^. Also, expression of the preadipocyte factor-1 (pref-1) continually decreased during adipogenesis in 3T3-L1 cells but transiently increased in MEFs^39^. As for PAT proteins, Plin5 mRNA levels drastically increased during adipogenesis of MEFs but not for 3T3-L1 cells^30^. Remarkably, Plin1 protein levels were diminished after lipolytic stimuli in MEFs adipocytes (Fig. 2F); however, they were almost constant in 3T3-L1 adipocytes (Supplementary Fig. S1A and ref. 40), although Plin2 localized on LDs in both types of adipocytes (Fig. 2D and Supplementary Fig. S1B). Taking these into consideration, it is plausible that the expression and regulation of proteins involved in lipolysis can be different between 3T3-L1 cells and MEFs. In this paper, we have shown that the decreasing rate of Plin1 after lipolytic stimuli was relatively higher in the sh-Plin2 group (Fig. 2F), although both Plin1 protein and intracellular TAG levels were indistinguishable before lipolytic stimuli between sh-control and sh-Plin2 groups. Hence, one of the mechanisms concerning how Plin2 regulates lipolysis in MEFs may be mediated by the change of Plin1 protein levels, which is supported by the evidence that both Plin1 and Plin2 can associate with CGI-58^41^. If it is presumed that certain genes involved in Plin1 protein degradation are not expressed in 3T3-L1 adipocytes, why Plin2 does not contribute to the regulation of lipolysis in the cells will be understood. It would be of great significance to investigate the physiological role of Plin2 and its related proteins during lipolysis in MEFs and then compare it with 3T3-L1 cells, which could eventually lead, not only to a better understanding of lipolysis but also the promotion of the value of MEFs as an *in vitro* adipocyte model.

We assumed that revealing the expressional regulation of Plin2 would greatly add to our understanding of the physiological role of Plin2 in adipocytes. As a result, we found that Plin2 in the cytosol but not the LD fraction was susceptible to ubiquitination and protein degradation (Figs 2, 4 and 5). On the other hand, it is well known that polyubiquitination to lysine residues on a target protein is important for its degradation by the ubiquitin-proteasome system^24^,^25^. However, we found that the mutant murine Plin2 in which 29 lysine residues were all substituted for alanine was still subjected to protein degradation (Supplementary Fig. S2), suggesting that Plin2 is not ubiquitinated through the classical pathway mediated by lysine residues. Additionally, we carried out a knockdown experiment with shRNA to downregulate the expression of atrophin-1-interacting protein 4 (AIP4), which is reported to act as Plin2 E3 ligase^42^, but Plin2 protein levels were not affected by AIP4 knockdown (unpublished data). Contrary to our finding that Plin2 is ubiquitinated in the cytosol (Fig. 5E), the report indicated that Plin2 was ubiquitinated on LDs, suggesting that cytosolic Plin2 is subjected to ubiquitination by an E3 ligase other than AIP4.

We further focused on the molecular mechanism for murine Plin2 protein degradation and found that second and third alanine residues in the N-terminal were essential for its degradation (Fig. 6). It is widely known that N-terminal residues of a target protein can be subjected to post-translational modification and subsequent polyubiquitination, and this system is called the N-end rule^35^,^36^. An amino acid sequence of Plin2 following methionine is, however, alanine (Fig. 6E), not glutamine, glycine, or cysteine, each of which are a classical consensus sequence for protein degradation through the N-end rule pathway. On the contrary, an atypical N-end rule was recently proposed in yeast and also mammalian cells^43^,^44^,^45^. According to the reports, N-terminal amino acids with a small side chain such as alanine, valine, serine, and threonine can be acetylated prior to ubiquitination and the following protein degradation. N-terminal sequences of Plin2 are widely conserved among species (Fig. 6E) that fit with the consensus sequence of this new N-end rule. We adopted the A (Ala) to D (Asp) mutants to verify the mechanism of Plin2 protein degradation because the second Asp is neither a typical nor atypical consensus sequence for the N-end rule, and the second amino acid of the Plin2 mutant (7–425) was Asp that was less susceptible to protein degradation. However, it should be noted that an A to D mutation introduces a negative charge and its protein structure could be affected accordingly, which might eventually lead to resistance to protein degradation. We believe that this issue will be resolved once the link between protein degradation and post-translational regulation of Plin2 is elucidated. We have preliminarily found that the first methionine in N-terminal Plin2-C-Flag was eliminated and the second exposed alanine was acetylated using mass spectrometry (unpublished data). Although it is not clear whether Plin2 is degraded through the new N-end rule pathway, the chances are high that the fate of Plin2 undergoes significant change at least through post-translational modifications. Through an examination of their roles and mediators such as E3 ligase, N-terminal acetyltransferase, and kinase, one can expect that the molecular mechanism and the physiological role of Plin2 in adipocytes will be further revealed.

In summary, we provided evidence that Plin2 protein is stabilized in response to lipolytic stimuli and can enhance lipolysis in MEF adipocytes. Furthermore, we showed that Plin2 is susceptible to proteasomal degradation in the cytosol but not on LDs, which is critically mediated by N-terminal alanine residues. It has already reported that Plin2 can associate with ATGL and/or CGI-58^41^,^46^, which are positive regulators of lipolysis. By elucidating the functions of Plin2-associated proteins, the whole picture of lipolysis becomes much more obvious, which is a pivotal physiological response essential for systemic energy homeostasis.

## Methods

### Reagents

Insulin, dexamethazone, BSA, MG-132, forskolin, lactacystin, E-64d, cycloheximide, anti-Flag antibody and a protease inhibitor cocktail were purchased from Sigma-Aldrich. IBMX and pioglitazone were from Wako Chemicals. ALLN and OAs were from Nacalai Tesque. Anti-PPARγ and anti-ubiquitin antibodies were from Santa Cruz Biotech. Anti-Plin1 and anti-Plin2 antibodies were from Progen. Anti-GFP and anti-actin antibodies were from Abcam and Chemicon, respectively.

### Plasmids

A retroviral expression plasmid for Flag-tagged mouse Plin1 was prepared as described previously^31^. Lentiviral expression plasmids for flag-tagged mouse Plin2 was similarly constructed by the insertion of the PCR fragment with full length or various mutants of Plin2 into CSII-EF-MCS-IRES2-Venus (RIKEN). Lentiviral expression plasmids for short hairpin RNA (shRNA) of mouse Plin2 or a control were constructed by recombining CS-RfA-EG (RIKEN) with pENTR4-H1 (RIKEN) inserted by oligonucleotide DNA. The target sequences were as follows: Plin2, 5′-GGAAGGATTTGATATGGTT-3′; control (Scramble II Duplex from Dharmacon), 5′-GCGCGCTTTGTAGGATTCG-3′.

### Mice and tissues

Wild-type and Plin1 deficient mice on a C57BL/6 background were obtained from the Jackson Laboratory. Epididymal, subcutaneous, and brown fat tissues as well as liver were harvested from these mice. The Institutional Animal Care and Research Advisory Committee at the University of Tokyo approved all animal procedures and the experiments were carried out in accordance with the committee’s guidelines.

### Preparation of MEFs and SVCs

Primary MEFs and SVCs were isolated from embryos at day 13.5 post coitum and subcutaneous fat pads of C57BL/6 mice, respectively. The preparation methods followed those described previously^47^.

### Cell culture

HEK293-T cells, NIH-3T3 cells, MEFs, and SVCs were cultured in Dulbecco’s modified Eagle’s medium (DMEM) with 10% fetal bovine serum (FBS), 100 unit’s/mL penicillin, and 100 μg/mL streptomycin. Plat-E cells^48^ were maintained in DMEM supplemented with 10% FBS, 100 units/mL penicillin, 100 μg/mL streptomycin, 10 μg/mL blasticidin, and 1 μg/mL puromycin. Adipocyte differentiation for MEFs and SVCs were processed as described previously^47^. All cultures were performed at 37 °C in 95% humidity with 5% CO_2_.

### Lentiviral and retroviral infection

Methods for the production and infection of each virus were followed as previously described^49^.

### Subcellular fractionation

Cells were homogenized on ice in buffer A (10 mM HEPES-KOH (pH 7.4), 250 mM sucrose, 1 mM EDTA, 50 μM ALLN, and a protease inhibitor cocktail) using 30 strokes of a 1 mL syringe with a 25G needle. The cell homogenates were centrifuged at 12,500 × g for 10 min to remove larger organelles. The supernatants were adjusted to 500 mM sucrose and layered in a centrifugation tube beneath a 0–500 mM sucrose gradient. After centrifugation at 100,000 × g for 1 h, up to 20 fractions were collected in equal amounts from the top. The top three fractions preferentially included LDs and are defined as the LD fractions.

### LD formation by BSA-conjugated OAs

To obtain the LD formation medium, DMEM with 1% BSA and 500 μM OAs was vigorously vortexed for 30 sec and subjected to sonication using an ultrasonic bath sonicator (BRANSON). After being filtrated through a 20 μm filter, the culture medium of NIH-3T3 cells was replaced by the medium.

### Immunoprecipitation and Western blot analysis

Cell proteins were extracted in RIPA buffer (50 mM Tris-HCl pH 8.0, 150 mM NaCl, 1% Triton X-100, 0.5% deoxycholate, 0.1% SDS, and a protease inhibitor cocktail). The lysates were then processed to immunoprecipitation and/or Western blot analysis as described previously^50^.

### Quantitative RT-PCR

Total cellular RNA was extracted from the cells using an RNA preparation kit (RNeasy Mini Kit, QIAGEN), and the following reverse transcription was performed using a High-Capacity cDNA Reverse Transcription Kit (Applied Biosystems). All mRNA levels were quantified using fluorescence real-time PCR on an ABI PRISM 7000 using TaqMan Gene Expression Assays (Applied Biosystems). The 18s rRNA-protein transcripts were used as an internal control to normalize mRNA levels of each gene.

### Confocal image analysis

Plin1+/+ or Plin1−/− MEFs differentiated into adipocytes on glass chamber slides were fixed with 4% paraformaldehyde in phosphate-buffered saline (PBS) (−) for 15 min at room temperature. After permeabilized with 0.2% TritonX-100 in PBS (−) for 5 min, the cells were treated with a blocking solution [2% BSA in PBS (−)]. The cells were then stained with BODIPY 493/503 (10 μg/mL, Molecular Probes) and an anti-Plin2 antibody (1:400), followed by Cy3-conjugated donkey anti-Guinea pig IgG (1:400, Jackson ImmunoResearch Laboratories). Fluorescence staining was then visualized using an Olympus FV500 confocal microscope.

### TAG measurements

The cells were washed with PBS (−), and lipids were extracted by hexane in 2-propanol (3:2, v/v). The amounts of intracellular triglyceride were determined using the Triglyceride E-test Wako (Wako) and normalized to the amounts of total cellular protein determined using a BCA protein assay (Pierce), according to each manufacturer’s instructions.

### Lipolysis induction and glycerol measurements

Lipolysis was induced by replacing the culture medium with DMEM with 2% BSA and 10 μM forskolin or 0.5 mM IBMX. After medium replacement, the cells, and the supernatants were separately recovered at each time point. The amounts of released glycerol were determined by mixing a free glycerol reagent (SIGMA) with the supernatants, according to the manufacturer’s instructions. The quantitative values were normalized to the corresponding total cellular protein levels measured by a BCA protein assay (Pierce).

### Oil Red O staining

After cells were fixed with 4% paraformaldehyde in PBS (−) for 1 h at room temperature, they were stained with Oil Red O solution [0.5% Oil red O in 2-propanol: milliQ = 3:2 (v/v)] for 1 h at room temperature.

### Statistical analysis

The results obtained in this study are presented as means ± S.D. Data were evaluated using the Student’s t-test for two groups. Results were considered to be significant when P < 0.05 and/or <0.01.

## Additional Information

**How to cite this article**: Takahashi, Y. *et al.* Perilipin2 plays a positive role in adipocytes during lipolysis by escaping proteasomal degradation. *Sci. Rep.* **6**, 20975; doi: 10.1038/srep20975 (2016).

## Supplementary Material

Supplementary Information
