# Supplementary material for: Perilipin2 plays a positive role in adipocytes during lipolysis by escaping proteasomal degradation
Source: Sci Rep. 2016 Feb 15;6:20975. doi: 10.1038/srep20975 (PMC4753471; doi:10.1038/srep20975)
Supplement: Supplementary Information [file srep20975-s1.pdf]

Supplementary Information

Perilipin2 plays a positive role in adipocytes during lipolysis by escaping proteasomal degradation

Yu Takahashi[1, *, †], Akihiro Shinoda[1, *, †], Haruhiko Kamada[2, 3], Makoto Shimizu[1], Jun Inoue[1] & Ryuichiro Sato[1, ‡]

[1]Department of Applied Biological Chemistry, Graduate School of Agricultural and Life Sciences, The University of Tokyo, Tokyo, Japan
[2]Laboratory of Biopharmaceutical Research, National Institute of Biomedical Innovation, Osaka, Japan
[3]The Center for Advanced Medical Engineering and Informatics, Osaka University, Osaka, Japan
*These authors contributed equally to this work.
[†]Present address: Japan Tobacco Inc., Central Pharmaceutical Research Institute, 1-1 Murasaki-cho, Takatsuki, Osaka, Japan
[‡]Correspondence and requests for materials should be addressed to R. S. (e-mail: aroysato@mail.ecc.u-tokyo.ac.jp)

Supplementary Fig. S1

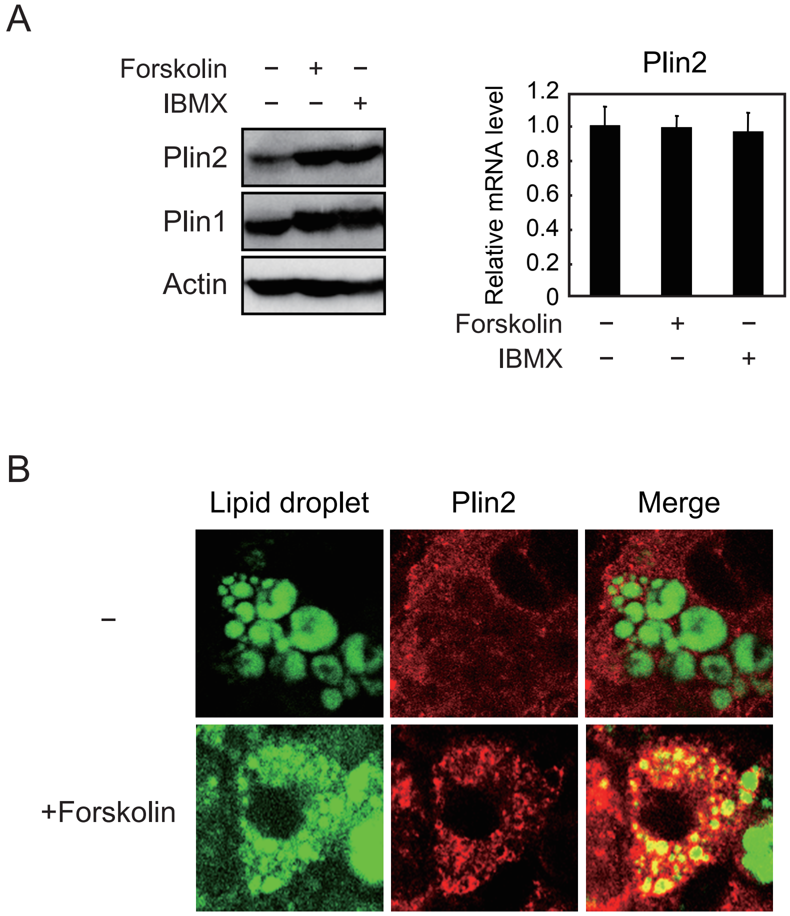

Supplementary Fig. S1. Plin2 is stabilized at the protein level and localize on LDs after lipolytic stimuli in 3T3-L1 adipocytes. (A) 3T3-L1 cells differentiated into adipocytes for 7 days were treated with 10 μM forskolin or 500 μM IBMX for 12 h. Each protein level from whole cell extracts was determined using Western blot analysis (left). The Plin2 mRNA level was determined using quantitative RT-PCR and normalized to 18s rRNA levels. These data represent the means ± S.D. The assay was performed in triplicate (right). (B) 3T3-L1 cells differentiated into adipocytes for 8 days were treated with 10 μM forskolin for 24 h. The cells were stained with BODIPY 493/503 (green, LDs) and anti-Plin2 antibody (red), and then underwent confocal image analysis as described in the Material and Methods.

Supplementary Fig. S2

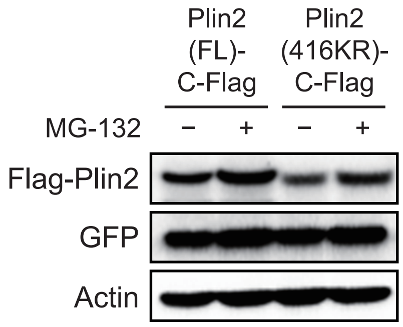

Supplementary Fig. S2. Lysine residues are not critical for protein degradation of murine Plin2. NIH-3T3 cells infected with either an Plin2-C-Flag or an Plin2(416KR)-C-Flag (All 29 lysine residues were substituted for alanine) lentiviral expression vector were treated with 10 μM MG-132 for 12 h. Each protein level from whole cell extracts was detected using Western blot analysis with anti-Flag, anti-GFP, and anti-actin antibodies.
